# Echoes of Support: A Qualitative Meta-Synthesis of Caregiver Narratives in Lung Cancer Care

**DOI:** 10.3390/healthcare12080828

**Published:** 2024-04-14

**Authors:** Panagiota Tragantzopoulou, Vaitsa Giannouli

**Affiliations:** 1 School of Social Sciences, University of Westminster, 115 New Cavendish St., London W1W 6UW, UK; 2 School of Medicine, Aristotle University of Thessaloniki, 54124 Thessaloniki, Greece; vaigia@auth.gr

**Keywords:** cancer care, lung cancer, caregivers, qualitative methods, systematic review

## Abstract

Lung cancer stands as one of the prevalent cancers, impacting both men and women globally. Family caregivers, deeply involved in the care of individuals affected by this disease, often endure heightened distress and struggle to navigate the manifold challenges associated with caregiving. Understanding the intricate experiences and challenges of caregivers in the realm of lung cancer care is critical, given its profound impact on their well-being and the quality of patient care. This study aimed to comprehensively examine and synthesize qualitative data concerning caregiver experiences within the context of lung cancer. Six databases were systematically searched for studies with qualitative findings relevant to caregivers and lung cancer. Seventeen studies were included, and findings were reviewed and synthesized. The main challenges identified were: ‘Information accessibility’, ‘Dual roles and family dynamics’, ‘Coping with emotional challenges and uncertainty’, and ‘Need for support networks’. These findings underscore the profound challenges faced by caregivers, shedding light on the substantial impact of cancer on their well-being and functionality. Moreover, the study accentuates the pressing need for tailored support systems that can address the emotional toll and information needs of caregivers. This emphasis on supportive interventions is vital to enhance the quality of care and overall well-being for both patients and caregivers within the lung cancer care continuum.

## 1. Introduction

Lung cancer, a prevalent form of malignant tumor, poses a substantial risk to human health. With the progress made in diagnostic and treatment techniques for this condition, alongside the expansion of the aging population, there has been a shift from inpatient to outpatient care [1,2]. This transition underscores the pivotal role of caregivers. Often comprising relatives, partners, or intimate friends, these caregivers share a deep personal bond with the patient, fulfilling a multifaceted role from aiding in daily tasks to providing emotional, social, and financial support [2]. However, this support often burdens caregivers, involving not just physical tasks but also emotional strain as they prioritize the patient’s needs over their own [3].

Compared to individuals facing other types of cancer, lung cancer patients commonly deal with a myriad of health issues, resulting in a diminished quality of life and poorer survival rates even with early detection [4,5]. The burden of this struggle extends to caregivers, who endure significant psychological distress and compromised physical health. Frequently taking on these responsibilities, caregivers often deal with a sense of inadequacy in meeting the patient’s evolving needs, especially as the illness progresses [6]. Previous qualitative studies have underscored significant challenges, including shifts in family dynamics, uncertainties regarding the future, and the essential need for information regarding treatment options and disease prognosis [7].

The combined weight of caregiving duties and the typically aggressive nature of lung cancer have profoundly adverse effects on the psychological, social, and spiritual facets of caregivers’ lives [8,9], resulting in declining psychological well-being and reduced quality of life. Previous studies have highlighted that the burden associated with caregiving for lung cancer patients correlates with higher chances of stress-related conditions such as depression and insomnia [10,11]. Furthermore, this burden can impede work performance and daily activities, potentially heightening the risk of social isolation [11].

Various interventions have emerged to assist caregivers, with a predominant emphasis on psychoeducational approaches and skill building aimed at enhancing caregiver capabilities in patient care [12,13]. A recent systematic review examining 22 studies assessing psychological interventions for caregivers of lung cancer patients revealed that a majority of these interventions, especially those emphasizing communication and incorporating multiple components, yielded improvements across diverse outcomes [14]. Although not always statistically significant, these interventions consistently showed enhancements in various areas including burden, distress, anxiety, depression, overall quality of life, self-efficacy, and coping skills [14]. However, only a limited number of interventions have specifically targeted fulfilling caregiver needs [12,13]. While several interventions have demonstrated positive impacts on psychological well-being [15,16], further research is essential to ascertain the most effective strategies and their integration into regular caregiving practices.

The objective of this review was to comprehensively investigate and consolidate insights into the caregiving experiences within the context of lung cancer patients. This particular group of caregivers has been recognized as prone to encountering psychological challenges and a reduced quality of life stemming from the demanding nature of caregiving responsibilities. By delving into the lived experiences of these caregivers, we aim to illuminate the specific support and interventions crucial for enhancing their quality of life and well-being. Such insights can enable caregivers to better support not only themselves but also the patients under their care.

## 2. Materials and Methods

We conducted a meta-synthesis of qualitative research, employing the methods outlined by Sandelowski and Barroso [17]. We consolidated, synthesized, and analyzed qualitative narratives detailing the experiences and challenges encountered by lung cancer care providers. Meta-syntheses represent comprehensive amalgamations that generate fresh insights from collective findings, surpassing the individual contributions [18]. They offer a comprehensive and holistic comprehension of a specific event or experience, going beyond isolated perspectives [18,19]. The study was not registered on PROSPERO due to disparities in methodology and objectives between systematic reviews and qualitative meta-synthesis, making PROSPERO registration less suitable for our study. Instead, our approach aligns more closely with established guidelines and frameworks for conducting qualitative synthesis. Given the dynamic and interpretive nature of the qualitative synthesis approach, which may not conform to PROSPERO’s predefined protocols, we chose not to register, prioritizing transparency through clear documentation over PROSPERO registration. Finally, the enhancing transparency in reporting the synthesis of qualitative research (ENTREQ) statement was employed to enhance the reporting of the meta-synthesis (Appendix A).

### 2.1. Search Strategy

The search strategy utilized terms (‘lung cancer’, ‘family caregiver’, ‘informal caregiver’, ‘caregiver’, ’caregiving experiences’, ‘caregiver support’, and ‘qualitative’) curated by the authors following an initial scoping search centered on studies regarding lung cancer and patient care. The strategy was tailored for application across six databases (PubMed, PsycINFO, CINAHL, Embase, Medline, and Web of Science). Aside from the database searches, we conducted a comprehensive review of each study’s references to identify additional the pertinent literature. Inclusion criteria encompassed qualitative studies involving caregivers of lung cancer patients who shared their experiences in providing care and were published in English between the years 2000 and 2023. Mixed-methods studies that reported the qualitative data separately were also included. Exclusion criteria comprised the literature reviews, editorials, letters, conference abstracts, and unpublished dissertations. Additionally, studies lacking at least one illustrative quote delineating carers’ experiences were excluded. The literature reviews, editorials, and letters were excluded because the focus of this study was on original research with novel findings. Conference abstracts were excluded as they often do not include quotes or provide clear data necessary for in-depth analysis. The grey literature, including unpublished dissertations, was excluded to ensure the inclusion of peer-reviewed studies meeting established quality standards.

### 2.2. Quality Assessment

The Critical Appraisal Skills Program (CASP) was implemented to assess the quality of the studies that fitted the inclusion criteria (Appendix B). The CASP qualitative checklist comprises 10 questions accompanied by structured instructions, systematically guiding the assessment of each study across various criteria such as validity, significance, and clarity. Each question was rated on a scale of zero to two: zero denoting ‘cannot tell’, one for ‘no’, and two for ‘yes’. Each study was screened and rated by the authors independently as per the guidelines outlined in the Critical Appraisal Skills Program (2018). Our primary aim in the quality appraisal process was to investigate the contributions of the studies to our research objectives and to gauge the validity of their outcomes. 

### 2.3. Qualitative Meta-Synthesis Methods

**Figure 1:**
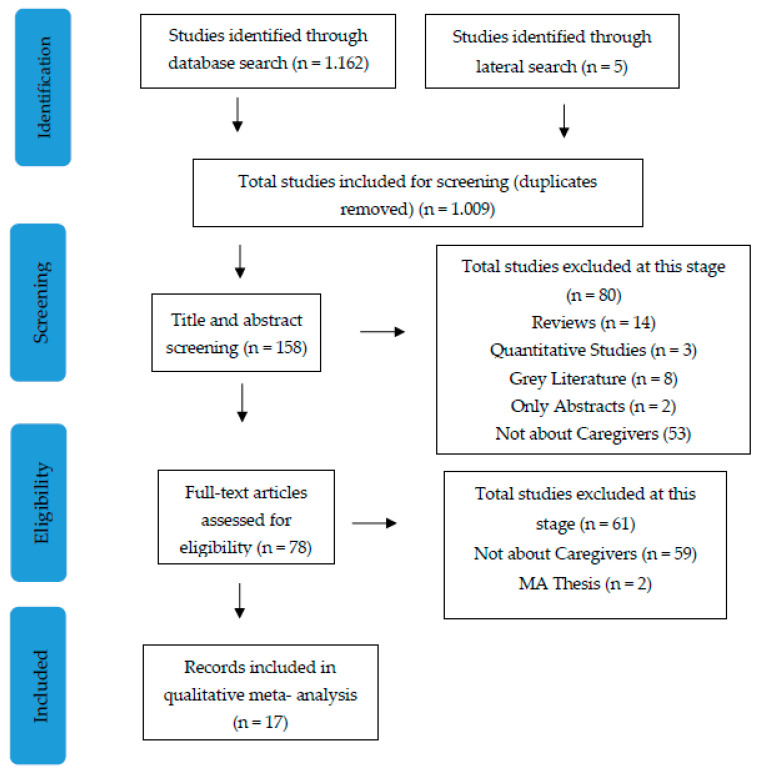
Overview of the selection process (PRISMA flow diagram).

We adopted Sandelowski and Barroso’s [17] method of integrating analysis, aiming to facilitate the identification of patterns and deviations within the data. This approach comprises six distinct steps: initiating the synthesis, reviewing the literature, evaluating findings, categorizing findings, amalgamating findings into meta-summaries, and amalgamating findings into a meta-synthesis. Firstly, an exhaustive literature search was conducted using systematic and iterative methods, including both database searches and hand-searching techniques (Figure 1). This process also included backward and forward citation searching, to ensure comprehensive coverage of the relevant literature. All the identified studies were screened, and duplicates were removed. In managing the literature, we utilized a reference management tool to organize and track the articles identified during the literature review process. Specifically, we employed Mendeley to store, organize, and manage the citations retrieved from electronic databases. This tool facilitated efficient citation management, enabling us to systematically track and retrieve relevant studies for further analysis. Next, the quality of the included studies was appraised with the use of CASP, focusing on both individual and comparative evaluation. Following this, analysis techniques such as classifying findings and meta-summarizing were employed. Findings from each study were extracted, entered into NVivo, and grouped, with abstract summaries created and effect sizes calculated as applicable. Finally, the synthesis output consisted of meta-summaries, which involved categorizing and organizing the findings into main themes and subthemes. Both authors reviewed and discussed the meta-summaries, noting similarities and differences. In cases of disagreement between the two authors during the evaluation process, a third reviewer was consulted to reach a consensus. This additional step ensured thoroughness and objectivity in the selection of articles for inclusion. Following the discussion and review of the caregivers’ direct quotes and the interpretations of these quotes, findings were synthesized into a meta-synthesis. Meta-synthesis transcends mere summarization by integrating findings through various methods, including comparisons and reciprocal translation. Reciprocal translation, in particular, is essential for preserving the distinctiveness of primary findings, achieved by blending in vivo concepts with imported ones from prior studies Ultimately, the meta-synthesis stage offered a novel interpretation and experimentation with innovations of findings, leading to a comprehensive understanding of the qualitative evidence. 

## 3. Results

### 3.1. Characteristics of Included Studies

The meta-synthesis encompasses 17 studies that were published between 2008 to 2023, showcasing a global perspective with studies originating from diverse regions. Among these, the US (n = 7) contributed the highest number of studies, followed by the UK (n = 2), Australia (n = 2), and Canada (n = 1), Germany (n = 1), Indonesia (n = 1), Ireland (n = 1), the Netherlands (n = 1), and China (n = 1). In total, the amalgamated studies involved 259 caregivers, collectively shedding light on the nuanced experiences within this caregiving domain.

Primarily qualitative in nature, the majority of studies (n = 16) were solely qualitative in approach, while one study adopted a mixed-methods design. Data collection relied on semi-structured interviews and focus groups, facilitating in-depth exploration and understanding of caregivers’ perspectives and challenges. The richness of these studies was enhanced by varied analytical approaches, encompassing thematic analysis, content analysis, grounded theory, and the structured framework of Creswell’s seven-step analysis. These diverse methodologies allowed for a comprehensive exploration of caregivers’ experiences, capturing multifaceted insights and nuances across different cultural contexts and caregiving landscapes. A comprehensive breakdown of study characteristics and pertinent details can be found in Table 1.

The qualitative meta-analysis identified four key themes, each shedding light on the multifaceted experiences of caregivers in the context of lung cancer. Effect sizes were calculated for each theme by dividing the number of primary studies featuring the designated theme by the total count of primary studies containing that specific finding. This approach offers a valuable perspective on the prominence of each theme within the literature, thereby enhancing our comprehension of the challenges faced by caregivers. In qualitative research, effect size plays a pivotal role in transcending mere description, enabling researchers to delve deeper into the substantive implications of their findings. In this study, the calculation of effect sizes revealed critical insights into the prevalence and significance of identified themes across the existing literature. For instance, the effect size for the first theme, ‘*Information accessibility*’, was 53%, suggesting that 9 out of 17 studies contributed to this overarching theme. Similarly, the second theme, ‘*Dual roles and family dynamics*’, exhibited an effect size of 47%, indicating the involvement of 8 studies out of 17 in exploring this aspect. Furthermore, the third theme, ‘*Coping with emotional challenges and uncertainty*’, demonstrated an effect size of 65%, with 11 out of 17 studies addressing this dimension. Finally, the fourth theme, ‘*Need for support networks*’, showed an effect size of 41%, underscoring the contribution of 7 out of 17 studies to this thematic area. These effect sizes not only quantify the prevalence of each theme but also provide valuable insights into the relative emphasis and significance attributed to different aspects of the phenomenon under investigation. We will delve into each theme, presenting the primary findings and utilizing quotes from the primary studies to exemplify them. 

### 3.2. Information Accessibility

In many studies, caregivers highlighted the lack of vital information about potential symptoms, treatments, and changes in the survivor’s condition post-hospitalization, leading to feelings of unpreparedness and uncertainty [20]. Hospital appointments were deemed crucial for these caregivers, focusing primarily on disease-related details such as treatment options, prognosis, and symptom management. Family caregivers actively participate in these visits, yet some hesitate to ask questions due to fear of burdening healthcare providers or perceiving their reluctance to address queries. This hesitancy was echoed in one caregiver’s statement:

“*We have a kind of reticence to ask our treating specialist questions. They do not like it when you ask a question*.”

[26]

In most studies, caregivers shared that effective communication with healthcare providers should involve clear, understandable information, fostering an open environment for questions, demonstrating expertise, and utilizing visual aids to explain complex medical details [21,22,24]. However, when direct communication fell short, caregivers turned to the internet, despite challenges in accurately interpreting complex medical information. The online space offered accessibility and anonymity, granting them the freedom to seek information at any hour. Internet-based platforms (e.g., familiar websites, social media platforms, discussion boards, and web-based patient portals) empowered caregivers to express themselves openly and eased anxiety compared with waiting for periodic specialist consultations:

‘*I think it is very good to be able to ask a physician questions online. It’s a smaller step to take than calling or talking to your treating specialist*.’

[26]

### 3.3. Dual Roles and Family Dynamics

Caregivers in cancer care often find themselves juggling multiple roles, undertaking various responsibilities simultaneously. The management of day-to-day tasks emerges as a prominent challenge for these caregivers. Many studies underscored the significant burden posed by practical obligations such as coordinating medical appointments, administering medications, handling financial matters, and arranging transportation. Ensuring timely and comprehensive care for their loved ones becomes demanding, as caregivers navigate complex healthcare systems and deal with insurance-related complexities. One caregiver mentioned that they have to coordinate everything:

“*getting him to the medical appointments, getting him his medication, and then also dealing with the financial aspects of it. Making sure that all his disability forms are filled in so he can get his check on time*.”

[30]

These responsibilities not only require substantial time and effort but also contribute to feelings of anxiety and frustration, particularly when faced with unexpected issues like delays in insurance claim processing or cancelled transportation to medical appointments. In Seibel’s et al. [35] study, it was highlighted that caregivers notably prioritized reshaping and adjusting their roles and relationship dynamics within the family’s day-to-day life, primarily due to the enduring effects of the situation. This frequently resulted in a demanding balancing act—serving both as a pillar of support and as someone personally affected:

“*[the situation due to the disease] stresses you out of course …. Psychologically and, I would also say, physically. Because you … must help more than usual. And then you are just doubly challenged*.”

[35]

Some even expressed reaching their own emotional or mental thresholds amidst these challenges. Few studies frequently depicted caregivers in diverse roles encompassing coordination, providing moral support, and offering practical care. The patient’s health status often pressured caregivers into assuming roles such as managing domestic activities or fulfilling both parental roles for their children. Balancing caregiving duties alongside other responsibilities posed a significant challenge, often leading to role shifts and adjustments:

“*Yes, I finally gave in … (the participant laughed), yes, I even washed the clothes and everything what housewife usually did because she was too weak, lack of energy … thank God I helped*.”

[36]

### 3.4. Coping with Emotional Challenges and Uncertainty

Most studies highlight that caregivers face significant emotional challenges and a pervasive sense of uncertainty when caring for individuals with cancer [23,32]. Among these challenges, emotional strain emerged as the most prevalent issue for informal caregivers:

“*It’s overwhelming. So, I try not to think about it, like when you feel this stuff coming on, all these questions, and they all congregate at once, you know. Especially when it’s 4 AM in the morning when you’re laying in bed, and there’s all these questions*.”

[25]

Sources of this strain included managing the care recipient’s emotions, assuming complete responsibility for their care, grappling with the unpredictable nature of the disease and its outcomes, dealing with the care recipient’s symptoms and complex issues, and sacrificing personal time. Caregivers also experienced emotional difficulties in supporting patients who were themselves undergoing emotional changes. Many caregivers mentioned their efforts to alleviate anxiety and fear in their loved ones, often by providing physical presence, engaging in routines, and offering distractions from negative thoughts and feelings:

“*I’m trying to occupy him so he doesn’t think… I make him go shopping with me, or I tell him, ‘let’s go for a ride. I don’t want to stay in the house.’ Or we’ll go in the backyard and sit under a tree and just talk*.”

[30]

Feelings of tension, mental stress, and anxiety were widespread, particularly before follow-up appointments due to the fear of cancer recurrence and re-experiencing the illness. Given the emotional challenges, caregivers resorted to various coping strategies. In a few studies, participants confirmed that they employed relaxation techniques such as breathing exercises, cognitive coping methods such as adjusting expectations, and meditation:

“*I do that meditation tape before bed. That seems to help me a lot in trying to sleep. Because it calms me down*.”

[25]

Some caregivers mentioned seeking solace in church and prayers, finding comfort in the belief that God was in control of the future:

“*And then we became very religious now. Every night we pray the rosary. And every Wednesday we go to church*.”

[25]

Uncertainty loomed large, especially concerning the obstacles they might encounter. Considering reducing or stopping work hours heightened their concerns about financial support for the patient. This uncertainty extended beyond sacrifices made in the present to apprehensions about future financial constraints and work–life balance. The lack of control over their lives and future led them to adopt a day-to-day approach, fostering a sense of unease without the ability to plan beyond the immediate present. Participants also expressed a sense of loss, feeling that cancer had taken away their dreams:

‘*Yes, today not tomorrow. I could never look forward, not not look forward but I don’t think about what’s going to happen tomorrow. I always think about what’s going to happen today. I wake up, how is she*?’

[33]

### 3.5. Need for Support Networks

Many participants relied on informal support networks such as family, friends, and community groups for emotional assistance during caregiving [34]. However, they struggled to find resources that addressed their unique challenges in balancing roles and managing finances. Caregivers expressed a sense of neglect in receiving formal support, feeling overlooked and unacknowledged in their own needs. This was highlighted in a participant’s statement:

“*No support for me at all. Zero, I would say. I don’t think anyone has ever in the whole process asked me how I was coping with it. Like we don’t count. Like people don’t think that we’re affected by it*.”

[27]

In a few studies, when discussing additional support requirements, suggestions were made for emotional assistance, such as caregiver meet-ups or patient advocates addressing the patient’s emotional journey [29]. One participant expressed the challenge of not knowing what could be done to address feeling ‘run-down’ as a mother and caregiver:

‘*I don’t know what to do with the situation. If I was having a bad day, I’d basically keep it to myself*.’

[28]

In a few studies, caregivers mentioned using formal support groups such as a care team or support groups to aid with emotional challenges. While some caregivers reported utilizing formal support groups, others found them distressing due to the overwhelming and often tragic stories shared, leading to increased stress. Caregivers described being negatively influenced by such support groups, as reading or listening to ‘extremely depressing’ or ‘tragic’ stories of other families coping with lung cancer could induce more stress:

“*We are going to forbid her from going back, because when you go to a place for support, and you come back and you’re more depressed than when you went, I… do not take that brand of support*.”

[31]

Other reasons for non-utilization of certain services included the perception that they were unnecessary or unhelpful for the care recipient’s mood, the inconvenience caused by the care recipient’s illness hindering extra assistance, and personal time constraints faced by caregivers:

‘*I … probably wouldn’t have time to attend anything or talk to anybody*.’

[28]

## 4. Discussion

This qualitative meta-synthesis delved into the recent literature, offering insights into the experiences of caregivers for lung cancer patients. To our knowledge, this represents the first meta-synthesis focusing on the caregivers’ experiences within the realm of lung cancer. This specific caregiving demographic holds significant importance, given the prevalence of lung cancer as one of the most commonly diagnosed cancers. As this disease continues to affect a growing number of individuals, understanding the challenges and experiences of caregivers becomes increasingly vital. Insight into the care provision for lung cancer patients can profoundly shape the nature of required support, offering invaluable assistance to those aiding individuals battling this illness.

The existing literature has consistently highlighted the pivotal role of internet-based resources in supporting caregivers of lung cancer patients, particularly in their quest for disease-related information, treatment options, prognosis, and symptom management [37,38]. These resources encompass familiar websites, social media platforms, discussion boards, and web-based patient portals, which caregivers frequently turn to for answers to their questions and concerns. Despite the abundance of online information, caregivers often perceive a scarcity of resources tailored to their needs, indicating a persistent gap in available support systems. While acknowledging the significance of internet use in cancer caregiving, as underscored by prior studies [37,38], our review delves deeper into a crucial aspect that warrants attention. It illuminates caregivers’ reliance on online resources not solely due to convenience, but also because of apprehension or reluctance in consulting healthcare providers directly. This dual reliance reflects caregivers’ complex needs—to grasp the nuances of cancer, understand treatment options and medical terminologies, and navigate the uncertain terrain of disease prognosis. Moreover, our findings emphasize the vital role of healthcare providers in supporting caregivers. While online resources offer a wealth of information, they cannot replace the personalized guidance and reassurance provided by healthcare professionals. Thus, there exists a critical need for healthcare providers to invest time in supporting caregivers, offering tailored guidance, and explaining the intricacies of the disease and its management. Cancer caregivers value healthcare providers who demonstrate attentiveness and responsiveness to their communication preferences while being considerate and adaptive to their unique communication needs, fostering a person-centered communication style [39]. Delivering healthcare services that cater to the unique needs of patients and their caregivers is crucial for enhancing positive care outcomes and shaping perceptions of care quality [40]. This approach forms the cornerstone of patient-centered care, emphasizing the importance of personalized and attentive healthcare delivery.

Our study sheds light on the multifaceted emotional challenges experienced by caregivers, elucidating the prevalent presence of heightened anxiety, mental stress, and fear. These findings align with prior research, which has consistently documented elevated levels of anxiety and depression among individuals in similar caregiving roles [10,11]. The emotional turmoil reported by caregivers predominantly emanates from the unpredictable trajectory of the disease and the pervasive uncertainty regarding future outcomes. Moreover, our investigation underscores the compounding effect of dual caregiving roles and shifts in family dynamics on caregivers’ emotional well-being, a phenomenon also noted in previous studies [30,35,36]. Importantly, our research unveils the proactive measures adopted by caregivers to mitigate distressing emotions. While previous studies have discussed some of these strategies, the synthesis of the findings in our study reveals the range of strategies employed. Strategies such as engaging in breathing exercises and employing cognitive coping methods emerged as prevalent approaches in our meta-synthesis. This adaptive coping behavior underscores caregivers’ resilience and proactive stance in managing their emotional well-being amidst the demanding responsibilities of caregiving. By drawing parallels with the existing literature, our findings contribute to a deeper understanding of the emotional landscape of caregiving and emphasize the significance of tailored support interventions for this vulnerable population.

Previous findings have underscored the indispensable role of familial, social, and community networks as primary pillars of support for caregivers [34]. Additionally, Hendriksen et al.’s [25] study identified the influence of religion and church as significant factors in caregivers’ coping mechanisms. This observation resonates with prior qualitative research, which has documented caregivers’ reliance on their faith for strength and solace during their caregiving journey [41]. Moreover, studies have consistently highlighted the potential benefits of spirituality and religion in facilitating a sense of ‘meaning making’ amidst the challenges of cancer [42]. However, it is crucial to note that, while spirituality and religion may not directly contribute to positive psychosocial adjustment, they serve as vital coping mechanisms, aiding individuals in navigating the complexities of cancer diagnosis and treatment [43]. By contextualizing these findings within the broader literature, our meta-synthesis offers insights into the nuanced interplay between social support networks and religious coping strategies in the caregiving context.

This role of coping mechanisms becomes particularly relevant in the context of caregiving interventions. Previous studies have evaluated interventions for caregivers, revealing their efficacy in reducing caregivers’ psychological distress and burden, while enhancing their preparedness for caregiving [15,16]. In contrast to these findings and despite the reported positive outcomes, our study highlights the underutilization of these interventions by caregivers. This meta-synthesis provides a distinctive contribution to the literature by investigating the reasons behind caregivers’ reluctance to engage with these interventions and services. Findings from this synthesis unveiled several factors contributing to the underutilization of interventions. Apart from citing a shortage of personal time as a limiting factor, caregivers expressed reservations about certain interventions, such as support groups, which were perceived as exacerbating their stress levels. Support groups might inadvertently amplify stress levels for caregivers. This could occur if the support group dynamics or discussions trigger heightened emotions or if the caregiver feels overwhelmed by the experiences shared. Further, the interventions offered might not align with the specific needs or preferences of certain caregivers. Delivering care that aligns with individual values involves blending conventional standardized care methods with personalized approaches that cater to the diverse and varied needs of caregivers [44]. Recognizing the heterogeneous nature of caregivers’ needs is pivotal in this process. By grasping the significance of factors pivotal to an individual’s health and overall well-being, we establish the groundwork for pinpointing significant care outcomes and avenues to harmonize care with their unique set of values [44].

This review presents both strengths and limitations. It effectively underscores the significance of recognizing caregivers’ roles and investigates the diverse challenges commonly faced by this demographic. Notably, the utilization of the CASP quality assessment framework substantially enhanced the rigor and dependability of the included studies. The comprehensive evaluation categorized each of the 17 studies as being of medium to high quality. However, a recurrent issue identified by the authors pertained to the consideration given to the relationship between researchers and participants. The review suggests that forthcoming studies should provide detailed information regarding this aspect. Nonetheless, beyond the identified limitations, there are several additional considerations to acknowledge. Firstly, while our meta-synthesis offers valuable insights into the experiences of caregivers for lung cancer patients, the geographical and cultural diversity of the included studies may be limited. This could potentially restrict the generalizability of our findings to populations beyond those studied. Despite our efforts to conduct a thorough search across multiple databases, there remains a possibility of missing relevant studies. This limitation could arise from variations in indexing practices across databases or the inclusion of studies published in languages other than English. Finally, an acknowledged limitation lies in the review’s exclusive focus on caregivers of lung cancer patients. While cancer variations may share commonalities in patient presentations, caregivers of diverse cancer types might encounter distinct challenges. Consequently, future research should aim to address the specific needs and challenges that caregivers in various cancer contexts may confront.

### Future Directions

As we navigate the landscape of caregiving for lung cancer patients, it becomes increasingly evident that formalized support programs tailored specifically for caregivers are an imperative necessity. These programs should be meticulously designed to address the multifaceted challenges caregivers encounter, encompassing the profound life changes, the multitude of responsibilities, and the unpredictable trajectories of the disease. Failure to adequately support caregivers ultimately compromises the well-being and preparedness of patients in their battle against lung cancer [31]. Therefore, the integration of structured support initiatives becomes not just beneficial but essential for a holistic approach to patient care.

Hospitals and healthcare institutions can play a pivotal role by establishing dedicated support teams, adept at alleviating the anxiety stemming from the myriad of treatment options and complex medical processes. These support teams can serve as a bridge, offering clear explanations, simplifying medical jargon, and providing comprehensive information to those dealing with the overwhelming complexities of the disease. By doing so, reliance on potentially misleading internet information is reduced, mitigating the escalation of stress and misinformation [37]. Moreover, it is imperative to train healthcare providers to offer empathic support to caregivers while addressing their own needs. Equipping these professionals with the skills to understand and tend to the emotional, mental, and logistical needs of caregivers is crucial in fostering a supportive ecosystem for both patients and their primary caregivers.

However, recognizing that existing official programs and services might fall short in addressing the diverse needs of caregivers, scholars and healthcare professionals alike should engage in the development and rigorous testing of innovative interventions. For instance, tailored interventions like a comprehensive online resource hub offering personalized guidance, community forums, and expert advice could prove invaluable in alleviating caregiver responsibilities and reducing the burden of multiple roles.

## 5. Conclusions

The findings derived from this meta-synthesis underscore the profound challenges faced by caregivers in the realm of lung cancer care, illuminating their significant impact on the well-being of caregivers and their support for their loved ones battling the disease. The synthesized evidence vividly illustrates how shifts in family dynamics, the adoption of dual roles, and the pervasive uncertainty inherent in the cancer journey can profoundly disrupt the emotional equilibrium of individuals. These shifts, compounded by contextual challenges, including the intricacies of medical information, reluctance to seek clarifications from healthcare providers who might exhibit limited responsiveness, and the deficiency or inadequacy of formal support services, contribute to an amplified sense of stress and burden among caregivers. This study may inform the development of interventions to help caregivers. By embarking on this path of innovation and prioritizing caregiver support, we move closer to a healthcare landscape where both patients and caregivers receive the comprehensive support necessary to navigate the complexities of lung cancer, fostering resilience, and well-being throughout the journey.

## Figures and Tables

**Table 1:** Characteristics of included studies.

Author (Year)	Country	Caregiver Sample Size	Data Collection Method	Analytical Approach
Cochrane et al., 2022 [20]	Ireland	9	Semi-structured interviews	Thematic analysis
Kedia et al., 2015 [21]	USA	24	Focus groups	Grounded theory
Fitch, 2020 [22]	USA	4	Semi-structured interviews	Content analysis
Otty et al., 2023 [23]	Australia	19	Semi-structured interviews	Thematic analysis
Kedia et al., 2018 [24]	USA	24	Focus groups	Creswell’s 7-step analysis framework
Hendriksen et al., 2019 [25]	USA	10	Semi-structured interviews	Grounded theory
Schook et al., 2014 [26]	The Netherlands	20	Semi-structured interviews	Thematic analysis
Occhipinti et al., 2018 [27]	Australia	12	Semi-structured interviews	Thematic analysis
Lee et al., 2022 [28]	Canada	20	Semi-structured interviews	Content analysis
Ryan et al., 2008 [29]	UK	20	Semi-structured interviews	Thematic analysis
Mosher et al., 2013 [30]	USA	21	Semi-structured interviews	Thematic analysis
Mosher et al., 2015 [31]	USA	21	Semi-structured interviews	Thematic analysis
Xue et al., 2022 [32]	China	18	Semi-structured interviews	Content analysis
Shilling et al., 2017 [33]	UK	6	Semi-structured interviews	Thematic analysis
Keimweiss et al., 2023 [34]	USA	5	Semi-structured interviews	Thematic analysis
Seibel et al., 2023 [35]	Germany	17	Semi-structured interviews	Content analysis
Sihombing et al., 2019 [36]	Indonesia	9	Semi-structured interviews	Content analysis

## Data Availability

Not applicable.

## Appendix A. The ENTREQ Checklist

**Item**	**Guide and Description**	**Reported on** **Page #**
Aim	State the research question the synthesis addresses	2
Synthesis methodology	Identify the synthesis methodology or theoretical framework which underpins the synthesis, and describe the rationale for choice of methodology (e.g., meta-ethnography, thematic synthesis, critical interpretive synthesis, grounded theory synthesis, realist synthesis, meta-aggregation, meta-study, framework synthesis).	2–3
Approach to searching	Indicate whether the search was pre-planned (comprehensive search strategies to seek all available studies) or iterative (to seek all available concepts until theoretical saturation is achieved).	2–3
Inclusion criteria	Specify the inclusion/exclusion criteria (e.g., in terms of population, language, year limits, type of publication, study type).	3
Data sources	Describe the information sources used (e.g., electronic databases (MEDLINE, EMBASE, CINAHL, psychINFO, Econlit), the grey literature databases (digital thesis, policy reports), relevant organizational websites, experts, information specialists, generic web searches (Google Scholar), hand searching, reference lists) and when the searches were conducted; provide the rationale for using the data sources.	2–3
Electronic search strategy	Describe the literature search (e.g., provide electronic search strategies with population terms, clinical or health topic terms, experiential or social phenomena-related terms, filters for qualitative research, and search limits).	3–4
Study screening methods	Describe the process of study screening and sifting (e.g., title, abstract and full text review, number of independent reviewers who screened studies)	3–4
Study characteristics	Present the characteristics of the included studies (e.g., year of publication, country, population, number of participants, data collection, methodology, analysis, research questions).	5 (Table 1)
Study selection results	Identify the number of studies screened and provide reasons for study exclusion (e.g., for comprehensive searching, provide numbers of studies screened and reasons for exclusion indicated in a figure/flowchart; for iterative searching, describe reasons for study exclusion and inclusion based on modifications to the research question and/or contribution to theory development).	2–4 and Figure 1
Rationale for appraisal	Describe the rationale and approach used to appraise the included studies or selected findings (e.g., assessment of conduct (validity and robustness), assessment of reporting (transparency), assessment of content and utility of the findings).	3
Appraisal items	State the tools, frameworks, and criteria used to appraise the studies or selected findings (e.g., Existing tools: CASP, QARI, COREQ, Mays and Pope [30]; reviewer developed tools; describe the domains assessed: research team, study design, data analysis and interpretations, reporting).	3 and Appendix B
Appraisal process	Indicate whether the appraisal was conducted independently by more than one reviewer and if consensus was required.	3–4
Appraisal results	Present results of the quality assessment and indicate which articles, if any, were weighted/excluded based on the assessment and provide the rationale.	Appendix B
Data extraction	Indicate which sections of the primary studies were analyzed and how the data were extracted from the primary studies (e.g., all text under the headings “results/conclusions” were extracted electronically and entered into a computer software).	3–4
Software	State the computer software used, if any.	3–4
Number of reviewers	Identify who was involved in coding and analysis.	3–4
Coding	Describe the process for coding of data (e.g., line-by-line coding to search for concepts).	3–4
Study comparison	Describe how were comparisons made within and across studies (e.g., subsequent studies were coded into pre-existing concepts, and new concepts were created when deemed necessary).	3–4
Derivation of themes	Explain whether the process of deriving the themes or constructs was inductive or deductive.	3–4
Quotations	Provide quotations from the primary studies to illustrate themes/constructs, and identify whether the quotations were participant quotations or the author’s interpretation	6–8
Synthesis output	Present rich, compelling, and useful results that go beyond a summary of the primary studies (e.g., new interpretation, models of evidence, conceptual models, analytical framework, development of a new theory or construct).	6–11

## Appendix B. CASP Quality Assessment

**Author (Year)**	**1. Was** **There a** **Clear** **Statement** **of the Aims** **of the** **Research?**	**2. Is a** **Qualitative** **Methodology** **Appropriate?**	**3. Was the** **Research** **Design** **Appropriate** **to Address** **the Aims of** **the** **Research?**	**4. Was the** **Recruitment** **Strategy** **Appropriate** **to the Aims** **of the** **Research?**	**5. Was the** **Data** **Collected** **in a Way** **that** **Addressed** **the** **Research** **Issue?**	**6. Has the** **Relationship** **between** **Researcher and** **Participants** **been** **Adequately** **Considered?**	**7. Have Ethical** **Issues Been** **Taken into** **Consideration?**	**8. Was the** **Data** **Analysis** **Sufficiently** **Rigorous?**	**9. Is There** **a Clear** **Statement** **of** **Findings?**	**10. How** **Valuable Is** **the** **Research?**
Cochrane et al., 2022 [20]	Yes	Yes	Yes	Yes	Yes	Yes	Yes	Yes	Yes	Yes
Kedia et al., 2015 [21]	Yes	Yes	Yes	Yes	Yes	Cannot tell	Yes	Yes	Yes	Yes
Fitch, 2020 [22]	Yes	Yes	Yes	Yes	Yes	Cannot tell	Yes	Yes	Yes	Yes
Otty et al., 2023 [23]	Yes	Yes	Yes	Yes	Yes	Cannot tell	Yes	Yes	Yes	Yes
Kedia et al., 2018 [24]	Yes	Yes	Yes	Yes	Yes	Yes	Yes	Yes	Yes	Yes
Hendriksen et al., 2019 [25]	Yes	Yes	Yes	Yes	Yes	Yes	Yes	Yes	Yes	Yes
Schook et al., 2014 [26]	Yes	Yes	Yes	Yes	Yes	Cannot tell	Yes	Yes	Yes	Yes
Occhipinti et al., 2018 [27]	Yes	Yes	Yes	Yes	Yes	Cannot tell	Yes	Yes	Yes	Yes
Lee et al., 2022 [28]	Yes	Yes	Yes	Yes	Yes	Cannot tell	Yes	Yes	Yes	Yes
Ryan et al., 2008 [29]	Yes	Yes	Yes	Yes	Yes	Cannot tell	Yes	Yes	Yes	Yes
Mosher et al., 2013 [30]	Yes	Yes	Yes	Yes	Yes	Cannot tell	Yes	Yes	Yes	Yes
Mosher et al., 2015 [31]	Yes	Yes	Yes	Yes	Yes	Cannot tell	Yes	Yes	Yes	Yes
Xue et al., 2022 [32]	Yes	Yes	Yes	Yes	Yes	Cannot tell	Yes	Yes	Yes	Yes
Shilling et al., 2017 [33]	Yes	Yes	Yes	Yes	Yes	Cannot tell	Yes	Yes	Yes	Yes
Keimweiss et al., 2023 [34]	Yes	Yes	Yes	Yes	Yes	Cannot tell	Yes	Yes	Yes	Yes
Seibel et al., 2023 [35]	Yes	Yes	Yes	Yes	Yes	Cannot tell	Yes	Yes	Yes	Yes
Sihombing et al., 2019 [36]	Yes	Yes	Yes	Yes	Yes	Cannot tell	Yes	Yes	Yes	Yes

## References

[1] Siegel R., Ma J., Zou Z., Jemal A. (2014). Cancer statistics, 2014. CA. Cancer J. Clin..

[2] Hirdes J.P., Freeman S., Smith T.F., Stolee P. (2012). Predictors of caregiver distress among palliative home care clients in Ontario: Evidence based on the interRAI Palliative Care. Palliat. Support. Care.

[3] Schubart J.R., Kinzie M.B., Farace E. (2008). Caring for the brain tumor patient: Family caregiver burden and unmet needs. Neuro-oncology.

[4] Stigimura H., Yang P. (2006). Long-term survivorship in lung cancer: A review. Chest.

[5] Li J., Girgis A. (2006). Supportive care needs: Are patients with lung cancer a neglected population?. Psycho-Oncology.

[6] Kim Y., Shaffer K.M., Carver C.S., Cannady R.S. (2016). Quality of life of family caregivers 8 years after a relative’s cancer diagnosis: Follow-up of the National Quality of Life Survey for Caregivers. Psycho-Oncology.

[7] Krishnasamy M., Wells M., Wilkie E. (2007). Patients and carer experiences of care provision after a diagnosis of lung cancer in Scotland. Support. Care Cancer.

[8] Fujinami R., Sun V., Zachariah F., Uman G., Grant M., Ferrell B. (2015). Family caregivers’ distress levels related to quality of life, burden, and preparedness. Psycho-Oncology.

[9] Ullrich A., Ascherfeld L., Marx G., Bokemeyer C., Bergelt C., Oechsle K. (2017). Quality of life, psychological burden, needs, and satisfaction during specialized inpatient palliative care in family caregivers of advanced cancer patients. BMC Palliat. Care.

[10] Nightingale C.L., Steffen L.E., Tooze J.A., Petty W., Danhauer S.C., Badr H., Weaver K.E. (2019). Lung Cancer Patient and Caregiver Health Vulnerabilities and Interest in Health Promotion Interventions: An Exploratory Study. Glob. Adv. Health Med..

[11] Jassem J., Penrod J., Goren A., Gilloteau I. (2015). Caring for relatives with lung cancer in Europe: An evaluation of caregivers’ experience. Qual. Life Res..

[12] Northouse L.L., Katapodi M.C., Song L., Zhang L., Mood D.W. (2010). Interventions with Family Caregivers of Cancer Patients: Meta-Analysis of Randomized Trials. CA Cancer J. Clin..

[13] Ferrell B., Wittenberg E. (2017). A review of family caregiving intervention trials in oncology. CA. Cancer J. Clin..

[14] Kedia S., Collins A., Dillon P.J., Akkus C., Ward K.D., Jackson B.M. (2020). Psychosocial interventions for informal caregivers of lung cancer patients: A systematic review. Psycho-Oncology.

[15] Aubin M., Vézina L., Verreault R., Simard S., Desbiens J.-F., Tremblay L., Dumont S., Dogba M.J., Gagnon P. (2021). A randomized clinical trial assessing a pragmatic intervention to improve supportive care for family caregivers of patients with lung cancer. Palliat. Support. Care.

[16] Sun V., Raz D.J., Erhunmwunsee L., Ruel N., Carranza J., Prieto R., Ferrell B., Krouse R.S., McCorkle R., Kim J.Y. (2019). Improving family caregiver and patient outcomes in lung cancer surgery: Study protocol for a randomized trial of the multimedia self-management (MSM) intervention. Contemp. Clin. Trials.

[17] Sandelowski M., Barroso J. (2006). Handbook for Synthesizing Qualitative Research.

[18] Thorne S., Jensen L., Kearney M.H., Noblit G., Sandelowski M. (2004). Qualitative Metasynthesis: Reflections on Methodological Orientation and Ideological Agenda. Qual. Health Res..

[19] Sandelowski M. (2007). From Meta-Synthesis to Method: Appraising the Qualitative Research Synthesis Report. Reviewing Research Evidence for Nursing Practice.

[20] Cochrane A., Gallagher P., Dunne S. (2022). “You just need to learn”: A qualitative study on the information needs of family caregivers of people with lung cancer. Eur. J. Oncol. Nurs..

[21] Kedia S.K., Ward K.D., Digney S.A., Jackson B.M., Nellum A.L., McHugh L., Roark K.S., Osborne O.T., Crossley F.J., Faris N. (2015). ‘One-stop shop’: Lung cancer patients’ and caregivers’ perceptions of multidisciplinary care in a community healthcare setting. Transl. Lung Cancer Res..

[22] Fitch M.I. (2020). Exploring Experiences of Survivors and Caregivers Regarding Lung Cancer Diagnosis, Treatment, and Survivorship. J. Patient Exp..

[23] Otty Z., Brown A., Larkins S., Evans R., Sabesan S. (2023). Patient and carer experiences of lung cancer referral pathway in a regional health service: A qualitative study. Intern. Med. J..

[24] Kedia S., Ward K., Digney S., Jackson B., Collins A., Rugless Stewart F., Faris N., Roark K., Osarogiagbon R.U. (2018). Qualitative assessment of organizational barriers to optimal lung cancer care in a community hospital setting in the United States. J. Community Support. Oncol.

[25] Hendriksen E., Rivera A., Williams E., Lee E., Sporn N., Cases M.G., Palesh O. (2019). Manifestations of anxiety and coping strategies in patients with metastatic lung cancer and their family caregivers: A qualitative study. Psychol. Health.

[26] Schook R.M., Linssen C., Schramel F.M., Festen J., Lammers E., Smit E.F., Postmus P.E., Westerman M.J. (2014). Why do patients and caregivers seek answers from the Internet and online lung specialists? A qualitative study. J. Med. Internet Res..

[27] Occhipinti S., Dunn J., O’Connell D., Garvey G., Valery P., Ball D., Fong K., Vinod S., Chambers S. (2018). Lung Cancer Stigma across the Social Network: Patient and Caregiver Perspectives. J. Thorac. Oncol..

[28] Lee C.T., Gonsalves C.L., Gao-Kang J., Pickrell W.G., Barker R.F. (2022). Resource utilization among informal caregiver of lung cancer patients undergoing treatment. Patient Exp. J..

[29] Ryan P., Howell V., Jones J., Hardy E. (2008). Lung cancer, caring for the caregivers. A qualitative study of providing pro-active social support targeted to the carers of patients with lung cancer. Palliat. Med..

[30] Mosher C.E., Jaynes H.A., Hanna N., Ostroff J.S. (2013). Distressed family caregivers of lung cancer patients: An examination of psychosocial and practical challenges. Support. Care Cancer.

[31] Mosher C.E., Ott M.A., Hanna N., Jalal S.I., Champion V.L. (2015). Coping with physical and psychological symptoms: A qualitative study of advanced lung cancer patients and their family caregivers. Support. Care Cancer.

[32] Xue M., Chen X., Zhao H., Zhao Y., Li J., Chen W. (2022). Understanding the experiences of older caregivers of patients with lung cancer during palliative chemotherapy in China: A qualitative study. Support. Care Cancer.

[33] Shilling V., Starkings R., Jenkins V., Fallowfield L. (2017). The pervasive nature of uncertainty—A qualitative study of patients with advanced cancer and their informal caregivers. J. Cancer Surviv..

[34] Keimweiss S., Gurolnick A., Grant S., Burris J., Studts J., Lewis-Thames M. (2023). “Just give it to us straight!”: A qualitative analysis of midwestern rural lung cancer survivors and caregivers about survivorship care experiences. J. Cancer Surviv..

[35] Seibel K., Sauer B., Wagner B., Becker G. (2023). “Scanxiety” and a sense of control: The perspective of lung cancer survivors and their caregivers on follow-up—A qualitative study. BMC Psychol..

[36] Sihombing Y., Waluyo A., Yona S. (2019). The experience of caring for an advanced lung cancer spouse: Vulnerable journey of caregiving. Enferm. Clin..

[37] Thiessen M., Raffin Bouchal S., Tang P.A., Sinclair S. (2023). Navigating the Cancer Journey Using Web-Based Information: Grounded Theory Emerging From the Lived Experience of Cancer Patients and Informal Caregivers With Implications for Web-Based Content Design. JMIR Cancer.

[38] Kinnane N.A., Milne D.J. (2010). The role of the Internet in supporting and informing carers of people with cancer: A literature review. Support. Care Cancer.

[39] Washington K.T., Craig K.W., Parker Oliver D., Ruggeri J.S., Brunk S.R., Goldstein A.K., Demiris G. (2019). Family caregivers’ perspectives on communication with cancer care providers. J. Psychosoc. Oncol..

[40] Kwame A., Petrucka P.M. (2021). A literature-based study of patient-centered care and communication in nurse-patient interactions: Barriers, facilitators, and the way forward. BMC Nurs..

[41] Palmer Kelly E., Meara A., Hyer M., Payne N., Pawlik T.M. (2019). Understanding the Type of Support Offered within the Caregiver, Family, and Spiritual/Religious Contexts of Cancer Patients. J. Pain Symptom Manag..

[42] Breitbart W. (2002). Spirituality and meaning in supportive care: Spirituality- and meaning-centered group psychotherapy interventions in advanced cancer. Support. Care Cancer.

[43] Edwards A., Pang N., Shiu V., Chan C. (2010). Review: The understanding of spirituality and the potential role of spiritual care in end-of-life and palliative care: A meta-study of qualitative research. Palliat. Med..

[44] Tuzzio L., Berry A.L., Gleason K., Barrow J., Bayliss E.A., Gray M.F., Delate T., Bermet Z., Uratsu C.S., Grant R.W. (2021). Aligning care with the personal values of patients with complex care needs. Health Serv. Res..

